# Symptomatic congenital cytomegalovirus disease following non-primary maternal infection: a retrospective cohort study

**DOI:** 10.1186/s12879-016-2161-3

**Published:** 2017-01-05

**Authors:** Eran Hadar, Elizabeta Dorfman, Ron Bardin, Rinat Gabbay-Benziv, Jacob Amir, Joseph Pardo

**Affiliations:** 1 Helen Schneider Hospital for Women, Rabin Medical Center, 39 Zabotinski St., Petach-Tikva, 49100 Israel; 2 Sackler Faculty of Medicine, Tel Aviv University, Tel Aviv, Israel; 3 Pediatrics C Department, Schneider Children’s Medical Center of Israel, Petach-Tikva, Israel

**Keywords:** Cytomegalovirus, Non-primary, Secondary, Diagnosis, Congenital, Risk factors

## Abstract

**Background:**

Scarce data exist about screening, diagnosis and prognosis of non-primary Cytomegalovirus (CMV) during pregnancy. We aimed to examine antenatal diagnosis of maternal non-primary CMV infection and to identify risk factors for congenial CMV disease.

**Methods:**

Retrospective cohort of 107 neonates with congenital symptomatic CMV infection, following either primary (*n* = 95) or non-primary (*n* = 12) maternal CMV infection. We compared the groups for the manifestations and severity of congenial CMV disease, as well as for possible factors associated with the risk of developing CMV related infant morbidity.

**Results:**

Disease severity is not similar in affected newborns, with a higher incidence of abnormal brain sonographic findings, following primary versus non-primary maternal CMV infection (76.8% vs. 8.3%, *p* < .001). Symptomatic congenital CMV disease following a non-primary infection is more frequent if gestational hypertensive disorders and/or gestational diabetes mellitus have ensued during pregnancy (33.3% vs. 9.9%, *p* <0.038), as well as if any medications were taken throughout gestation (50% vs. 16.8%, *p* <0.016). CMV-IgM demonstrates a low detection rate for non-primary maternal infection during pregnancy compared to primary infection (25% vs. 75.8%, *p* = 0.0008).

**Conclusion:**

Non-primary maternal CMV infection has an impact on the neonate. Although not readily diagnosed during pregnancy, knowledge of risk factors may aid in raising clinical suspicion.

## Background

Cytomegalovirus (CMV) is a common congenital infection, diagnosed among 0.3-2% of newborns [1]. The prevalence and severity of congenital CMV infection varies according to the timing of infection, the presence of preexisting immunity and other contributing factors [2]. Secondary, or non-primary, maternal CMV infection occurs when prior maternal immunity exists. It can emerge following latent CMV reactivation or by a new viral strain reinfection [3]. Following a non-primary maternal infection the vertical transmission rate of CMV is reported to be 0.2-1% [4] and congenital symptomatic disease is diagnosed in less than 1%, among fetuses infected in-utero [5]. Moreover, congenital CMV has a clinical spectrum ranging from asymptomatic infection to a severe disease, including multiple possible manifestations of petechiae, jaundice, hepatitis, chorioretinitis, microcephaly, growth restriction, hepatosplenomegaly; as well as short and long term squelae of hearing loss and neurodevelopmental deficits [6]. It is debatable whether prior immunity infers complete or partial protection against CMV related morbidity [7, 8], while others have reported that disease manifestations, severity and long term prognosis are similar between primary and non-primary infections [9–11].

The rate of congenital infection following non-primary maternal CMV infection is extremely low compared to the incidence subsequent to primary infection [12, 13]. Nevertheless, non-primary maternal CMV infection has been previously reported as a cause of congenital symptomatic disease [14, 15]. In the United States, only 25% of infants with congenital CMV infection were attributable to primary maternal infection, while 75% were born to mothers with non-primary infections, following either reactivation or reinfection with different viral strains [16]. Moreover, in a recent population-based prediction model, it was estimated that non-primary infections account for the majority of CMV-related hearing losses [17].

Paucity of reports exist on appropriate tools to screen and diagnose non-primary gestational CMV and to detect women who are at risk for delivering CMV symptomatic infants, following a non-primary infection. Therefore, we aimed to examine the antenatal diagnosis of maternal non-primary CMV infection and to identify antenatal risk factors for congenial symptomatic CMV among neonates.

## Methods

We conducted a retrospective cohort study of all neonates that were referred for follow up due to congenital CMV infection. The study was approved by the Rabin medical center institutional review board.

### Study population

The study population included all neonates born from January 2006 to December 2013, who were referred for follow up due to congenital symptomatic CMV infection. The study was conducted at the Rabin medical center, which is a university-affiliated, tertiary medical center which serves as a referral center for both high risk pregnancies (Helen Schneider Hospital for Women) and pediatric patients (Schneider Children’s Medical Center of Israel).

Eligibility criteria for the study included all the newborns with a positive urinary culture or PCR for CMV, taken shortly after birth and up to two weeks following delivery. Those with insufficient data as to antenatal and/or neonatal care and serological data were excluded from the study, as well as those newborns with a congenital asymptomatic infection. The included population was sub-divided to symptomatic neonates following either non-primary maternal CMV infection (study group) versus primary infection (control group).

Symptomatic newborns included those with any one of the following symptoms or signs - small for gestational age (SGA, defined as birthweight below the 3rd percentile for gestational age at delivery), chorioretinitis, abnormal brain sonography (defined as the presence of any of the following: calcification, periventricular hyperecogenicity, ventriculomegaly, subependymal cysts and lenticular-striated vasculopathy (LSV)), microcephaly (defined as age appropriate head circumference below two standard deviations), pathological audio screening, sensorineural hearing loss (SNHL) detected by abnormal brainstem-evoked response audiometry test (BERA) and that the treating physician has decided to start antiviral treatment for any one of the above mentioned signs and symptoms.

### Maternal and neonatal follow up

The diagnosis of primary maternal CMV infection was established according to the following criteria: women who seroconvert from negative to positive CMV-IgG during pregnancy; or if prior serology for CMV was not available - low CMV-IgG avidity and positive specific CMV-IgM were also considered diagnostic for primary maternal CMV infection. Non-primary CMV infection was considered when pre-gestational maternal immunity was established from previous laboratory results, i.e. positive CMV-IgG documented prior to pregnancy.

Mothers who were diagnosed during pregnancy with gestational CMV infection were offered prenatal diagnosis with amniocentesis. The procedure was performed beyond 21 completed weeks of gestation and at least 7 weeks after the presumed timing of maternal CMV infection. Following amniocentesis, if fetal infection was documented and pregnancy was not terminated, serial ultrasound (US) assessments were recommended every 3–4 weeks and magnetic resonance imaging (MRI) was suggested at 32–34 weeks. Fetal imaging results - by either MRI or US - were considered as abnormal, an related to CMV, if they included any one of the following findings: fetal growth restriction (defined as estimated fetal weight below the 3rdpercentile for gestational age), ventriculomegaly, microcephaly, central nervous system (CNS) calcification, periventricular hyperecogenicity, subependymal cysts, increased white matter signal and abnormal gyration or hyperechogenic bowel.

Congenital CMV infection in the newborn was established by a CMV positive urinary shell vial culture or PCR, preformed within 48 h after birth and up to 2 weeks following delivery. Neonates with a positive culture underwent a thorough evaluation, upon admission, comprised of: BERA, ocular funduscopy, brain ultrasound and blood work-up for complete blood count, liver enzymes and bilirubin. CNS sonographic signs related to neonatal CMV included: calcifications, periventricular hyperecogenicity, ventriculomegaly, pseudocysts and LSV. Neonates diagnosed with CMV infection after birth were followed at our clinic once a month until 3 months of age and every 3-6monthsthereafter. A full physical examination, including neurological and developmental assessments, was performed at each visit. Blood count was tested every other week up to 3 months of age and then during subsequent clinical visits. BERA was performed in the first month, and thereafter at least twice in the first year of life, with follow up periodic examinations every 6 months to age 2 years. Behavioral learning evaluation was preformed every 6 months up to age 4.

### Data extraction and definitions

We extracted maternal, fetal and neonatal data from medical charts, delivery records and computerized databases of laboratory and imaging data. The extracted data for the study included: maternal demographics, medical and obstetrical history, maternal occupation (high risk occupations for CMV exposure were defined as kindergarten teachers, day care workers and medical professionals), pregnancy complications (Hypertensive disorders, gestational diabetes, preterm delivery prior to 37 weeks of gestation and maternal febrile illness during pregnancy), use of medication throughout pregnancy and delivery outcome (mode and time of delivery, fetal gender, Apgar score and birthweight). Laboratory and imaging data was also extracted, including: complete maternal serologic data (IgG, IgM and IgG avidity), liver enzyme testing, amniocentesis and imaging (US and/or MRI) results for CMV obtained during pregnancy and neonatal follow up.

### Statistical analysis

Statistical analysis was performed using the Statistical Package for Social Science (SPSS, IBM software version 21.0, Chicago, IL, USA). Categorical variables were compared using Fischer’s exact or Pearson Chi-square tests. Continuous variables were analyzed by One Way Analysis of Variance (ANOVA). A probability value below 0.05 was considered significant.

## Results

A total of 173 newborns with proven congenital CMV infection were evaluated, of which six were excluded due to lack of antenatal data and 60 due to a neonatal asymptomatic infection. Therefore, 107 newborns with symptomatic congenital CMV disease were available for final analysis - 12 (11.2%) newborns were symptomatic following non-primary CMV maternal infection (study group) and 95 (88.8%) were affected following primary CMV maternal infection (control group).

The manifestations of the congenital CMV disease among the neonates, in each of the groups, are presented in Table 1. Our results demonstrate that symptomatic newborns following maternal non-primary CMV infection were significantly less likely to present an abnormal brain sonography compared to neonates with congenital CMV disease following primary CMV infection (8.3% vs. 76.8%, *p* < .001).

**Table 1:** CMV related findings among symptomatic neonates, following primary versus non-primary maternal infection

	Primary (*n* = 95)	Non-primary (*n* = 12)	*P* value
Small for gestational age	17 (17.9%)	1 (16.6%)	0.686
Chorioretinitis	5 (5.3%)	0	0.999
Abnormal brain sonography	73 (76.8%)	1 (8.3%)	<.0001
Microcephaly	5 (5.3%)	1 (8.3%)	0.904
Pathological audio screening test	18 (18.9%)	3 (25%)	0.7
Abnormal BERA			
At admission	32 (33.7%)	7 (58.3%)	0.117
At follow up	40 (42.1%)	7 (58.3%)	0.498

Data presented as n (%)

*CMV* Cytomegalovirus, *BERA* Brainstem Evoked Response Audiometry

### Maternal factors (Table 2)

**Table 2:** Maternal characteristics among symptomatic neonates, following primary versus non-primary maternal infection

	Primary (*n* = 95)	Non-primary (*n* = 12)	*P* value
Maternal age, years	29.12 ± 5.12	27.77 ± 4.60	0.452
Maternal chronic disease	14 (15.1%)	3 (27.3%)	0.382
High professional risk	19 (20%)	2 (16.7%)	0.999
Obstetrical history			
Parity	2.24 ± 1.21	2.16 ± 1.69	0.848
Live births	2.03 ± 0.93	2.0 ± 1.41	0.918
Abortions	0.21 ± 0.74	0.17 ± 0.39	0.847
Parity, with living children (≥1)	75 (78.9%)	7 (58.3%)	0.146
Complications during pregnancy	9 (9.9%)	4 (33.3%)	0.038
Medication use during pregnancy	16 (16.8%)	6 (50%)	0.016
Febrile disease at CMV diagnosis	31 (38.8%)	1 (8.3%)	0.103
Gestational age at CMV diagnosis:	19.50 ± 8.38	19.16 ± 13.51	0.928
1^st^ trimester	44 (46.3%)	3 (25%)	0.318
2^nd^ trimester	42 (44.2%)	1 (8.3%)	0.03
3^rd^ trimester	9 (9.5%)	2 (16.7%)	0.356
Maternal serology at CMV diagnosis			
Positive IgG	81 (85.3%)	12 (100%)	<.0001
IgG Seroconversion	79 (83.1%)	--	--
Positive IgM	72 (75.8%)	3 (25%)	0.0008
Low avidity IgG	33 (34.7%)	--	--

Data presented as n(%) or mean ± 1 Standard Deviation

*CMV* Cytomegalovirus

Women giving birth to a symptomatic newborn, following a non-primary CMV infection during pregnancy, were more likely to have medical complications during pregnancy (33.3% vs. 9.9%, *p* <0.038) and were more likely to have used medications during pregnancy (50% vs. 16.8%, *p* <0.016) compared to women delivering after a primary CMV infection. Serological diagnosis of maternal infection was established during pregnancy in only 3 women among the non-primary infection subgroup. We found that the likelihood of positive IgM-CMV was significantly lower (25% vs. 75.8%, *p* = 0.0008) among the non-primary subgroup compared to the primary subgroup, respectively.

### Fetal factors (Table 3)

**Table 3:** Fetal characteristics among symptomatic neonates, following primary versus non-primary maternal infection

	Primary (*n* = 95)	Non-primary (*n* = 12)	*P* value
Fetal gender			
Male (*n* = 56)	51 (53.7%)	5 (41.7%)	0.544
Female (*n* = 51)	44 (46.3%)	7 (58.3%)	0.544
Mode of delivery			
Vaginal delivery	64 (67.4%)	8 (66.7%)	0.999
Instrumental delivery	6 (6.3%)	2 (16.7%)	0.22
Cesarean delivery	25 (26.3%)	2 (16.7%)	0.726
Gestational age at delivery, weeks	38.92 ± 1.56	39.00 ± 2.00	0.882
Birth weight, grams	3070 ± 530	3167 ± 817	0.607
Neonatal head circumference, cm	33.71 ± 1.63	33.77 ± 1.93	0.909
Amniocentesis preformed during pregnancy:	47 (49.5%)	2 (16.7%)	0.035
Positive for CMV	42 (89.4%)	0	0.018
Abnormal findings on fetal US	11 (11.6%)	4 (33.4%)	0.114
Abnormal findings on fetal MRI	11 (11.6%)	0	0.608

Data presented as n(%) or mean ± 1 Standard Deviation

*CMV* Cytomegalovirus, *US* Ultrasound, *MRI* Magnetic Resonance Imaging

Mothers exposed to non-primary CMV infection were less likely to perform amniocentesis than those with primary infection (16.7% vs. 49.5%, *p* = 0.035). Also, the chance of positive CMV in amniotic fluid analysis among fetuses exposed to non-primary CMV infections was lower than those in the primary subgroup (0% vs. 89.4%. *p* = 0.018).

## Discussion

Our study was a retrospective cohort analysis of 107 newborns, stratified according to preexisting maternal immunity. Our main results indicate that disease manifestation and severity are not similar between groups; antenatal risk factors for a congenital CMV disease following a non-primary infection include the occurrence of pregnancy complications and the use of medication during pregnancy; and that CMV-IgM demonstrates low detection rate for non-primary maternal infection.

Previous studies have reported contradicting findings, whether CMV following non-primary infection is associated with a higher or lower incidence [18, 19] and severity [7–11] of congenital CMV. Boppana et al. [9], in a similarly designed study to our own, compared 47 symptomatic neonates - 12 following non-primary CMV and 35 following primary CMV infection. They demonstrated similar rates of CMV related symptoms (SGA, jaundice, hepatosplenomegaly, microcephaly, chorioretinitis and abnormal hearing screen) and comparable CMV disease severity among the groups. Townsend et al. [10] and Ross et al. [11] have shown, respectively, that hearing loss and overall long term sequelae have similar incidence in primary and non-primary CMV symptomatic newborns. Contradictory to these reports, Fowler et al. [8], comparing 125 neonates born following primary maternal infection vs. 64 neonates born following non-primary infection demonstrated that only those with primary infection were symptomatic at birth – including jaundice, petechiae, hepatosplenomegaly, SGA, microcephaly, hydranencephaky and death. Also, that late onset handicaps and squelae were apparent among 25% of neonates born following primary infection, versus only 8% in neonates of the non-primary CMV group. Our study partly supports all these findings, as we demonstrated similar rates of SGA, microcephaly, chorioretinitis, pathological audio screening test and BERA. However, we found higher rates of abnormal brain sonography in the primary CMV group, a sign which has not been evaluated specifically in previous studies.

The majority of current literature on the prediction of symptomatic CMV disease among infected fetuses is focused on the timing of maternal infection during gestation and the presence of abnormal findings in fetal imaging, as predictors of symptomatic congenital disease. Our study is the first to demonstrate that non-sonographic antenatal factors may be associated with an increased risk to deliver an affected newborn. Our results support the possibility that the occurrence of pregnancy complications - specifically hypertensive disorders and gestational diabetes - as well as the use of chronic medication during pregnancy is associated with an increased risk of delivering a CMV symptomatic newborn, following a non-primary CMV maternal infection. There is paucity of data on antenatal risk factors for symptomatic disease, and no data focusing on the subgroup of non-primary CMV. Boppana et al. [9], demonstrated no difference among primary versus non-primary groups, with regards to parity and the number of children of the infected mothers. Others [20] have reported that young age (below 20 years), non-white race and low-income status are associated with an increased risk for delivering a neonate exhibiting CMV related morbidity - however, no distinction was made with regard to maternal immunity status during pregnancy in this study. In a previously published study [21], we demonstrated that antenatal risk factors for CMV congenital disease following primary maternal infection may include - young maternal age and a high risk occupational exposure. Accordingly, we speculate that primary infection is related to external factors, conferring a higher exposure risk of the mother to new onset CMV infection, while a non-primary infection is associated with internal factors, exposing the mother to either reactivation or reinfection with CMV. Although the mechanism for such an association is unknown, we speculate that it is may be associated with some degree of interference to the immune system, leading to higher maternal susceptibility to latent viral reactivation. Although, for either primary or non-primary infection, the final common pathway is a higher viral load during gestation, allowing for greater viral inoculums to reach the placenta and fetus, consequently increasing the risk for symptoms at birth.

Serological diagnosis of non-primary CMV infection in the mother is problematic. Due to the asymptomatic nature of maternal CMV infection in the majority of cases, clinical suspicion of CMV infection rarely rises. Maternal CMV infection is usually diagnosed by routine screening in sero-negative women or may be suspected due to abnormal sonographic findings. In our cohort maternal serology was examined for the presence of CMV-IgM in only 6 cases. The trigger for serological testing was due to either febrile illness (*n* = 1), suspicious sonographic finding (*n* = 4) or routine testing (*n* = 1). Among those 6 mothers, CMV-IgM was positive in only half (50%). This rate was significantly lower than the rate of positive CMV-IgM (75.8%) among mothers in the primary infection. The low rate of positive CMV-IgM among mothers with non-primary CMV infection is supported by other small studies [22, 23]. Kyriazopoulou et al. [22] studied 32 women immunized to CMV with normal pregnancy course and no suspicious ultrasound findings. Although, none of the women had any serological evidence of maternal non-primary CMV infection, they detected 4 (12.5%) positive CMV results in amniotic fluid or fetal blood sampling. In another study, Zalel at al. [23] studied 6 pregnant women who were referred due to abnormal sonographic findings, suggestive of in-utero CMV related morbidity. All women were exposed to CMV prior to pregnancy and none had serological evidence of non-primary infection at workup. However, all women had positive CMV in amniotic fluid analysis. These studies, in concordance with our current study, demonstrate a low detection rate for non-primary CMV infection by CMV-IgM testing.

A major strength of our study is that in contrast to other studies, the immune status of all participating women was known and ascertained by documented serological examinations. Indeed, the study is limited due to its retrospective nature, with a limited number of participants, especially in the non-primary CMV group, where only symptomatic newborns were referred for follow-up. Such a referral is routinely recommended when suspected finding arise during pregnancy or at the immediate postpartum period due to abnormal sonographic findings, clinical findings and abnormal audio screening tests - this may explain the high rate of hearing loss in the study. Importantly, the primary CMV infection group includes only newborns to mothers who elected to continue their pregnancy despite proven in-utero infection or alarming findings on prenatal imaging. Due to lack of data we could not determine whether the non-primary infection was a result of reactivation or reinfection, both possible causes of congenital CMV [24], although no data exist whether one causes more serious morbidity than the other [7].

## Conclusion

The true incidence and impact of recurrent maternal CMV infection remains elusive, with questions as to risk factors, screening and diagnosis. However, it seems that the incidence of symptomatic non-primary infection is low and somewhat limited to pregnancies complicated by maternal morbidity. When such fetal-neonatal infection occurs, it might be with less severe sequelae compared to neonates following primary maternal infection. Our results bring to focus the importance of sonographic follow up during pregnancy, which may provide clues for the possibility of congenital symptomatic CMV fetal infection. When such relevant sonographic markers are detected, the absence of presumed serological evidence (i.e. positive IGM), should not prevent further diagnostic measures such as amniocentesis and further fetal imaging such as a detailed targeted sonography and MRI. This also raises the issue of newborn routine screening as a major tool for prompt and timely diagnosis of congenital CMV. Such early detection may allow workup, treatment and future explorations to prevent long term handicap. Undoubtedly, further large scale studies are needed, focusing on prevention of non-primary CMV among gravidas, fetal diagnosis during pregnancy and screening for congenital CMV among neonates, as well as possible interventions to prevent long term poor prognosis among congenitally infected neonates.

## Abbreviations

BERA: Brainstem-evoked response audiometry
CMV: Cytomegalovirus
CNS: Central nervous system
LSV: Lenticular striate vasculopathy
MRI: Magnetic resonance imaging
SGA: Small for gestational age
SNHL: Sensorineural hearing loss
US: Ultrasound
